# Acute Stress and an Electrolyte- Imbalanced Diet, but Not Chronic Hypoxia, Increase Oxidative Stress and Hamper Innate Immune Status in a Rainbow Trout (*Oncorhynchus mykiss*) Isogenic Line

**DOI:** 10.3389/fphys.2019.00453

**Published:** 2019-04-24

**Authors:** Leonardo J. Magnoni, Sara C. Novais, Ep Eding, Isabelle Leguen, Marco F. L. Lemos, Rodrigo O. A. Ozório, Inge Geurden, Patrick Prunet, Johan W. Schrama

**Affiliations:** ^1^CIIMAR – Centro Interdisciplinar de Investigação Marinha e Ambiental, Universidade do Porto, Matosinhos, Portugal; ^2^MARE – Marine and Environmental Sciences Centre, ESTM, Instituto Politécnico de Leiria, Peniche, Portugal; ^3^Aquaculture and Fisheries Group, Wageningen Institute of Animal Sciences, Wageningen University, Wageningen, Netherlands; ^4^Laboratoire de Physiologie et Génomique des Poissons, Institut National de la Recherche Agronomique, Rennes, France; ^5^Nutrition Metabolisme Aquaculture (NuMeA)- Institut National de la Recherche Agronomique (INRA), Saint-Pée-sur-Nivelle, France

**Keywords:** rainbow trout, dietary imbalance, metabolic capacity, fish homeostasis, chronic hypoxia, stressors

## Abstract

In aquaculture, fish may be exposed to sub-optimal rearing conditions, which generate a stress response if full adaptation is not displayed. However, our current knowledge of several coexisting factors that may give rise to a stress response is limited, in particular when both chronic and acute stressors are involved. This study investigated changes in metabolic parameters, oxidative stress and innate immune markers in a rainbow trout (*Oncorhynchus mykiss*) isogenic line exposed to a combination of dietary (electrolyte-imbalanced diet, DEB 700 mEq Kg^-1^) and environmental (hypoxia, 4.5 mg O_2_ L^-1^) challenges and their respective controls (electrolyte-balanced diet, DEB 200 mEq Kg^-1^ and normoxia, 7.9 or mg O_2_ L^-1^) for 49 days. At the end of this period, fish were sampled or subjected to an acute stressor (2 min of handling/confinement) and then sampled. Feeding trout an electrolyte-imbalanced diet produced a reduction in blood pH, as well as increases in cortisol levels, hepato-somatic index (HSI) and total energy content in the liver. The ratio between the lactate dehydrogenase (LDH) and isocitrate dehydrogenase (IDH) activities decreased in the liver of trout fed the DEB 700 diet, but increased in the heart, suggesting a different modulation of metabolic capacity by the dietary challenge. Several markers of oxidative stress in the liver of trout, mainly related to the glutathione antioxidant system, were altered when fed the electrolyte-imbalanced diet. The dietary challenge was also associated with a decrease in the alternative complement pathway activity (ACH_50_) in plasma, suggesting an impaired innate immune status in that group. Trout subjected to the acute stressor displayed reduced blood pH values, higher plasma cortisol levels as well as increased levels of metabolic markers associated with oxidative stress in the liver. An interaction between diet and acute stressor was detected for oxidative stress markers in the liver of trout, showing that the chronic electrolyte-imbalance impairs the response of rainbow trout to handling/confinement. However, trout reared under chronic hypoxia only displayed changes in parameters related to energy use in both liver and heart. Taken together, these results suggest that trout displays an adaptative response to chronic hypoxia. Conversely, the dietary challenge profoundly affected fish homeostasis, resulting in an impaired physiological response leading to stress, which then placed constraints on a subsequent acute challenge.

## Introduction

Farming fish involves following certain practices and procedures which may include handling, low water levels, confinement, and crowding (Conte, 2004). These procedures can act as stressors when habituation is not present (Pickering, 1993; Wendelaar Bonga, 1997; Bratland et al., 2010; Nilsson et al., 2012). Additionally, chronic sub-optimal rearing conditions may also give rise to stress responses if full adaptation is not displayed. However, the information available on existing conditions that may act as stressors during rearing, along with their interactions, is very limited. This is an area of increasing interest, as stressors have been linked to a reduction in both growth performance and immune condition; hampering health and welfare in fish (Portz et al., 2006).

One of the known existing factors of stress in cultured fish species is the persistent low oxygen concentration (chronic hypoxia). Nevertheless, its consequences are still poorly documented. In addition, long-term dietary imbalances affect fish homeostasis and energy balance. The induction of stress by dietary imbalances have been described in fish, including responses linked to partial or total replacement of fish meal and fish oil by alternative sources (Montero et al., 2003, 2008, 2010; Gómez-Requeni et al., 2004). Previous studies have shown that growth performance, feed intake and nutrient digestibility are altered when fish are fed electrolyte imbalanced diets (Dersjant-Li et al., 1999, 2000; Saravanan et al., 2013b; Magnoni et al., 2016). However, the induction of stress by this type of dietary imbalance is yet to be investigated in fish.

The dietary electrolyte balance (DEB) is defined as the sum of the mineral cations minus the sum of mineral anions present in the diet. Differences in DEB may occur when feed ingredients containing different quantities of cations (Na, K, Ca, and Mg) and anions (Cl and P) are included in the diet formulation (Patience and Wolynetz, 1990). It was previously shown that rainbow trout (*Oncorhynchus mykiss*) fed an electrolyte imbalanced diet (DEB 700) were energetically less efficient than those fed an electrolyte balanced diet (DEB 200). In spite of this change in energy balance, feed intake and growth performance were not affected (Magnoni et al., 2018).

The objective of this study was to determine whether electrolyte imbalanced diet may affect the ability of trout to cope with chronic hypoxia conditions. The assessment of physiological impacts of combined stress factors (diet and hypoxia) was analyzed in fish before and after applying an acute stressor (handling/confinement). This acute stress condition is commonly present in aquaculture, and was used to determine the physiological coping ability of the fish.

A set of plasma parameters related to stress and innate immune response were determined, as they are commonly associated with fish welfare. Additionally, several parameters were analyzed in liver and heart, since their energy use are altered when subjected to stress conditions (Wendelaar Bonga, 1997; Hermes-Lima et al., 2001), including changes in oxidative stress and metabolic response. An isogenic heterozygous family of rainbow trout was used as fish model, due to its genetic uniformity providing low intra-specific variability and high reproducibility.

## Materials and Methods

### Fish and Housing

The isogenic heterozygous family of rainbow trout (R23) obtained by crossing two homozygote isogenetic lines was produced by INRA/PEIMA (France) experimental fish facilities (Sadoul et al., 2015). Fish were housed in the Aquatic Metabolic Unit (AMU) tanks of Aquaculture and Fisheries group, Wageningen University, The Netherlands. Thirty rainbow trout (115.2 ± 2.0 g) were randomly assigned to each of the twelve experimental tanks (200 L). The tanks were connected to a water recirculation system consisting of a trickling filter, an oxygenation unit, a sump, a drum filter (Hydrotech 500^®^), and a cooling/heating system for maintaining uniform water quality throughout the study. The oxygenation unit maintained the DO levels by injecting oxygen into the water and was facilitated with separate automatic probes for the detection of water flow and oxygen consumption. Water temperature was set at 14 ± 1°C. Photoperiod was maintained at 12:12 (Light: Dark) with daybreak set at 07:00 h.

### Experimental Diets and Feeding

Two isoproteic (45% DM) and isoenergetic (22 kJ gDM^-1^) diets, floating pellets of 4 mm, were extruded by Research Diet Services (Wijk bij Duurstede, The Netherlands). Diets, upon arrival to the AMU at University of Wageningen, were stored in a room under controlled conditions during the trial. The two diets were formulated to provide a contrast in electrolyte content (DEB); 200 or 700 mEq Kg^-1^. This difference was created by adding different amounts of Na_2_CO_3_ and diamol (inert filler) in the diets. Fish were fed the experimental diets to apparent satiation, twice a day for 49 days. Ingredients and proximate composition of the experimental diets are included in Supplementary Table S1.

### Experimental Conditions

The difference in DO levels was induced by adjusting the rate of water flow into the tanks as described by Saravanan et al. (2013a). The water volume was kept constant at 200 L in all tanks. For the normoxia groups, the rate of water inflow into each tank was kept at 7.2 ± 0.0 L min^-1^ (mean ± SEM) with a mean water DO level of 10.2 ± 0.1 mg O_2_ L^-1^. The DO level in the outflowing water remained at 7.9 ± 0.1 mg O_2_ L^-1^. Hypoxia conditions were created by gradually reducing the rate of water inflow into the tank (2.2 ± 0.0 L min^-1^) with a DO level of 10.2 ± 0.1 mg O_2_ L^-1^ for the first 3 days after the start of the experiment. DO level in the outflowing water remained at 4.5 ± 0.1 mg O_2_ L^-1^ and was kept equal in all the hypoxic tanks. The DO level in the water outlet was considered to be equivalent to ambient DO level of the tanks, as differences between both DO levels (outlet versus inside the tank) shown in several previous experiments were negligible (<0.2 mg O_2_ L^-1^, e.g., Tran-Duy et al., 2008). The force of the evenly distributed water supplied by the inlet in each tank together with the swimming activity prevented the occurrence of water stratification within the tanks. The DO level applied in the hypoxia treatment is recognized as an environmental challenge, with the value decided based on the reported incipient DO level of 6.0 mg O_2_ L^-1^ for rainbow trout (Pedersen, 1987).

### Experimental Design

Employing a 2 × 2 design (DEB diets and DO levels), the experimental tanks (12) were divided into three experimental blocks, with four tanks randomly assigned within each of three blocks (*n* = 3 tanks per treatment). Fish were not fed on the day prior to sampling.

Fish from each experimental group were divided into 2 sub-groups for the application of a standardized handling/confinement protocol (acute stress) and posterior sampling. A control sub-group in which three fish per tank (9 per treatment) were sampled by reducing the exposure to potential stressors. Subsequently, a group of 3 fish per tank were netted and exposed to a confinement stress (2 min at a density of 200 kg/m^3^) and transferred back to their original tank (empty) for 1 h. Then fish from both experimental sub-groups were netted and euthanized for sampling. A sampling effect was prevented by sampling the tanks following the order tanks were installed in the room, starting with the control sub-group and with water from sampled tanks on flow through to prevent water re-entering the RAS circulation. A lethal dose of 2-phenoxy ethanol solution (1 mg/L) was used in all the sampling procedures.

Blood was drawn from the caudal region with a heparinized syringe. Blood pH was immediately measured (pH meter, WTW pH 320; pH electrode, WTW SenTix Sp). The duration of procedure (blood withdrawal and pH measurement) was strictly standardized to 1 min for all fish to minimize blood pH variation (Saravanan et al., 2013b). Blood was centrifuged at 3000 g for 20 min at 4°C, and plasma samples were frozen and stored for later analyses. Fish, liver, and heart were weighed and sampled. Samples were frozen in liquid nitrogen and stored at -80°C for further analyses.

### Plasma Metabolite Content

Lactate concentration in plasma was quantified using a commercial kit (LO-POD, Spinreatc, Sant Esteve de Bas, Spain). Glucose concentration in plasma was quantified using a commercial kit (GOD-POD, Spinreatc, Sant Esteve de Bas, Spain). An ELISA kit based on the competitive link between cortisol and related monoclonal antibodies was used to quantify cortisol levels in plasma of rainbow trout (RE 52061, IBL International, Hamburg, Germany). Validation of the kit for determination of cortisol in plasma of trout was performed. The intra- and inter-assay coefficient of variation in plasma samples were <9% and <10%, respectively. The linearity showed an *r*^2^-value of 0.96. Results from this validation indicated the suitability for the use of this kit to quantify changes in cortisol levels in rainbow trout. All measurements were performed in triplicates, following the recommendations provided by the manufacturers.

### Energy Balance in Tissues

Total protein, glycogen and lipids contents were measured spectrophotometrically in the tissue homogenates according to De Coen and Janssen (1997, 2003). Liver and heart tissues were homogenized in phosphate buffer (1/10 vol, 0.1 M, pH = 7.4). Proteins in the homogenates were precipitated with the addition of cold 15% trichloroacetic acid and incubated at -20°C for 10 min. After a centrifugation at 1000 g for 10 min, the resulting supernatant was used for glycogen quantification by adding phenol (5%) and H_2_SO_4_ (conc.). After 30 min incubation at 20°C, glucose was quantified by measuring the absorbance at 490nm. The protein pellet obtained after trichloroacetic acid precipitation was resuspended in NaOH (1N), incubated at 60°C for 30 min and then neutralized with HCl (1.67 M). The resuspended pellet was used to quantify total protein content by the Bradford (1976) method measuring absorbance at 600 nm, using bovine serum albumin as standard.

As for lipids extraction, chloroform, methanol, and Mili-Q water were added to homogenates in a 2:2:1 proportion, respectively. The organic phase was separated after centrifugation, treated with H_2_SO_4_ at 200°C, and used to quantify total lipid content at 400 nm, using tripalmitin as standard. Total protein, glycogen, and lipid content of each sample was expressed as mg per g of wet weight tissue. Total energy content was calculated as the sum of the protein, glycogen and lipids contents in each tissue, transformed into energetic equivalents using values for the enthalpy of combustion (24 kJ/g proteins, 17.5 kJ/g carbohydrates, and 39.5 kJ/g lipids), as described by De Coen and Janssen (1997), and expressed as kJ g^-1^ wet weight wet tissue.

An aliquot of homogenate was centrifuged for 5 min, at 3000 g (4°C) and the supernatant was used for lactate dehydrogenase (LDH) and isocitrate dehydrogenase (IDH) activity measurements. LDH was measured following the method described by Vassault (1983) and adapted to microplate. IDH was measured following the method described by Ellis and Goldberg (1971) with the adaptations of Lima et al. (2007). For the normalization by protein content, Bradford (1976) method was applied to quantify protein in the supernatant fraction, using bovine G-globulin as standard and reading absorbance at 600 nm. Energy consumption rate (Ec: measured through the electron transport system -ETS- activity) was performed according to the procedure described by De Coen and Janssen (1997, 2003). Measurements were performed at 25°C in triplicates using proper reaction blanks in a synergy H1 Hybrid Multi-Mode microplate reader (Biotek^®^ Instrument, Vermont, United States).

### Oxidative Stress Markers in Liver

Liver and heart samples were homogenized in phosphate buffer (1/10 vol., 0.1 M pH 7.4). Enzymatic analyses were all carried out with the reaction mixtures and homogenate dilution established in preliminary tests. Protein concentration was assayed in homogenates using bovine serum albumin as standard (Bradford, 1976). Lipid peroxidation (LPO) was determined by quantifying the presence of formed thiobarbituric acid reactive substances (TBARS) (Ohkawa et al., 1979). Glutathione reductase (GR) (EC1.8.1.7) and glutathione peroxidase (GPx) (EC 1.11.1.9.) were evaluated based on NADPH (Sigma, Portugal) oxidation at 340 nm (Mohandas et al., 1984; Cribb et al., 1989). Total glutathione (TG) and oxidized glutathione (GSSG) were evaluated by the formation of 5-thio-2-nitrobenzoic acid at 412 nm (Baker et al., 1990). Reduced glutathione (GSH) was calculated as the difference between TG and GSSG. Changes in absorbance were measured at 22°C in a Power-Wave^TM^ microplate spectrophotometer (BioTek Instruments), and reactions were performed in triplicates. Substrate was omitted in reaction blanks and background activity was subtracted from that measured in the presence of substrate.

### Innate Immune Parameters in Plasma

Immune status was assessed through the determination of key parameters, namely lysozyme, peroxidase and the alternative complement pathway (ACH_50_) activities. Lysozyme activity was evaluated according to Lie et al. (1986), employing hen egg white lysozyme (Sigma, Germany) as standard and *Micrococcus lysodeikticus* (0.5 mg mL^-1^; 0.05 M sodium phosphate buffer; pH 6.2) as bacterial suspension. Peroxidase activity (U mL^-1^ plasma) was determined based on the methodology described by Quade and Roth (1997). ACH_50_ activity was analyzed according to Sunyer and Tort (1995) using a concentration of 2.8 × 10^8^ cells mL^-1^ rabbit erythrocytes. The reciprocal of the plasma dilution giving 50% haemolysis of erythrocytes equals to one ACH_50_ unit.

### Measurements and Calculations

Weight gain (WG) was calculated as:

$$\begin{array}{c} \;\;{WG(g) = BW}_{f}{-\mathrm{BW}}_{i} \end{array}$$

where BW_i_ and BW_f_ are the initial and the final body weight of fish in the trial, respectively.

Feed intake (FI) was calculated as:

$$\begin{array}{c} \;{FI(gDM) = FI}_{\mathrm{TOT}}/n\times t, \end{array}$$

where FI_TOT_ is the total FI per tank over the experimental period corrected for dead fish and uneaten feed, n is the number of fish per tank and t is the experimental period (days).

Uneaten feed (pellets) were collected on the surface at the end of each feeding period (1 h). Pellets remaining on the bottom of the tank were collected by a decantation unit. All the uneaten pellets were counted and the amount calculated by taking and average weight of pellets in each diet production lot. The amount of feed was registered daily and accounted for in the feed intake calculation. No mortalities were recorded during the trial, except one fish fed the DEB 200 diet in hypoxia (98.9% survival).

Feed conversion ratio (FCR) was calculated as:

$$\begin{array}{c} \;FCR=FI/(BW_{f}-BW_{i}) \end{array}$$

Specific growth rate (SGR) was calculated as:

$$\begin{array}{c} \;SGR(\%BWday^{-1})=(lnBW_{f}-lnBW_{i})/time(days)\;\times \;100 \end{array}$$

The hepato-somatic index was calculated as:

$$\begin{array}{c} \;HSI(\%)=100\;\times \;[liverweight(g)/BW(g)] \end{array}$$

The cardio-somatic-index was calculated as:

$$\begin{array}{c} \;CSI(\%)=100\;\times \;[heartweight(g)/BW(g)] \end{array}$$

The effects of DEB 200 and DEB 700 on growth performance and feed intake of this trout isogenic line subjected chronic hypoxia or normoxia has been published in Magnoni et al. (2018). However, to assist with the interpretation of the results presented in the current study, growth performance and feed intake parameters were included as Supplementary Table S2.

### Statistical Analysis

Final body weight (BW_f_), weight gain (WG), and specific growth rate (SGR) were analyzed for the effects of the diet and DO levels along with their interactions by a two-way ANOVA analysis (triplicate tanks). All the other parameters in fish were analyzed for the effects of diet, DO levels and acute stress, along with their interactions by a three-way ANOVA analysis (*n* = 9). When differences in mean values were detected by the ANOVA analysis, *post hoc* tests were applied (Holm-Sidak method). In all these analyses, data sets were tested for normality (Shapiro–Wilk) and for equal variance (Levene’s test), and if these assumptions were not met, data transformation was applied (Natural logarithm). Statistical analysis was made using SigmaPlot 12.0 (Systat Software, Inc. 2011) and significant differences were considered for *P* < 0.05.

## Results

### Metabolic Markers in Blood and Plasma

Blood pH remained similar in fish reared at different DO levels (Table 1). Feeding trout an electrolyte-imbalanced diet (DEB 700) or subjecting the fish to acute stress induced a reduction on blood pH (*P* < 0.001).

**Table 1:** Effect of dietary electrolyte balance (DEB), dissolved oxygen levels, and acute stress on blood parameters of rainbow trout.

	DEB 200				DEB 700				Factors			Interactions			
	Normoxia		Hypoxia		Normoxia		Hypoxia							
	C	S	C	S	C	S	C	S	D	O	S	DOS	DO	DS	OS
pH	7.06 ± 0.2	6.99 ± 0.02	7.08 ± 0.02	7.02 ± 0.02	7.0 ± 0.01	6.95 ± 0.03	7.07 ± 0.02	6.92 ± 0.03	^∗∗^	ns	^∗∗^	ns	ns	ns	ns
Cortisol	6.63 ± 1.95	28.00 ± 4.88	6.58 ± 1.84	34.88 ± 8.50	11.66 ± 1.80	41.62 ± 4.52	8.55 ± 1.68	31.84 ± 6.35	^∗^	ns	^∗∗^	ns	ns	ns	ns
Lactate	0.26 ± 0.07	0.45 ± 0.11	0.31 ± 0.03	0.65 ± 0.13	0.27 ± 0.06	0.60 ± 0.10	0.29 ± 0.07	0.63 ± 0.05	ns	ns	^∗∗^	ns	ns	ns	ns
Glucose	5.40 ± 0.31	7.22 ± 0.60	5.39 ± 0.45	6.83 ± 0.47	5.34 ± 0.30	7.29 ± 0.16	5.31 ± 0.44	6.32 ± 0.47	ns	ns	^∗∗^	ns	ns	ns	ns

*Plasma cortisol levels in ng mL^*-1*^. Plasma lactate and glucose concentrations in mM. Values are mean of fish (n = 9)****±****SE. C, Control; S, Acute stress. Three-way ANOVA analysis results. D, Diet; O, dissolved oxygen levels; DOS, DO, DS, and OS, Interactions; ns, not significant P > 0.1; ^∗^P < 0.05; ^∗∗^P < 0.01.*

Plasma cortisol levels were increased in trout subjected to acute stress or when fed an electrolyte-imbalanced diet (*P* < 0.001 and *P* < 0.005, respectively). However, cortisol levels remained unaltered when fish were subjected to chronic hypoxia. Plasma lactate and glucose concentrations were increased in fish subjected to acute stress (*P* < 0.001) but remained similar when fed diets with varied DEB or when maintained at different DO levels.

### Energy Use in Tissues

The hepato-somatic index (HSI) of trout was 16% higher in fish fed the DEB 700 diet (*P* < 0.05) (Table 2). All the parameters related to energy content measured in liver of trout remained similar at different DO levels. However, total energy content in the liver was increased by 6% in fish fed the electrolyte-imbalanced diet (*P* < 0.05).

**Table 2:** Effect of dietary electrolyte balance (DEB), dissolved oxygen levels and acute stress on hepato-somatic index (HSI), protein, lipids, glycogen, and energy content in liver of rainbow trout.

	DEB 200				DEB 700				Factors			Interactions			
	Normoxia		Hypoxia		Normoxia		Hypoxia						
	C	S	C	S	C	S	C	S	D	O	S	DOS	DO	DS	OS
HSI	1.42 ± 0.22	1.31 ± 0.15	1.25 ± 0.17	1.39 ± 0.23	1.56 ± 0.18	1.73 ± 0.19	1.29 ± 0.13	1.81 ± 0.32	^∗^	ns	ns	ns	ns	ns	ns
Protein	61.87 ± 5.65	61.50 ± 2.61	61.82 ± 7.63	54.51 ± 4.56	59.35 ± 3.12	58.09 ± 3.34	61.54 ± 3.92	53.68 ± 6.41	ns	ns	ns	ns	ns	ns	ns
Lipids	24.47 ± 1.95	22.10 ± 1.17	25.33 ± 1.86	28.06 ± 2.89	26.37 ± 2.80	26.86 ± 3.31	23.43 ± 0.73	28.83 ± 3.46	ns	ns	ns	ns	ns	ns	ns
Glycogen	95.18 ± 14.62	74.50 ± 8.59	88.15 ± 14.31	103.74 ± 10.19	109.81 ± 6.32	105.52 ± 10.08	83.47 ± 7.69	112.72 ± 10.72	ns	ns	ns	ns	ns	ns	^∗^
Total energy	4.12 ± 0.13	3.65 ± 0.14	4.03 ± 0.16	4.23 ± 0.17	4.39 ± 0.13	4.30 ± 0.21	3.86 ± 0.82	4.40 ± 0.16	^∗^	ns	ns	ns	^∗^	ns	^∗∗^

*Protein, lipids and glycogen expressed as mg g^-1^ weight wet tissue. Total energy content expressed as kJ g^-1^ weight wet tissue. Values are mean of fish (n = 9)****±****SE. C, Control; S, Acute stress. Three-way ANOVA analysis results. D, Diet; O, dissolved oxygen levels; DOS, DO, DS, and OS, Interactions; ns, not significant P > 0.1; ^∗^P < 0.05; ^∗∗^P < 0.01.*

The cardiac-somatic index (CSI) of trout was reduced by 6% in fish subjected to hypoxia (*P* < 0.05) (Table 3). The energy stored as glycogen in the heart of trout was increased by 27% in fish reared in hypoxia (*P* < 0.01) and decreased by 26% when subjected to acute stress (*P* < 0.01).

**Table 3:** Effect of dietary electrolyte balance (DEB), dissolved oxygen levels and acute stress on cardiac-somatic index (CSI), protein, lipids, glycogen, and energy content in heart of rainbow trout.

	DEB 200				DEB 700				Factors			Interactions			
	Normoxia		Hypoxia		Normoxia		Hypoxia							
	C	S	C	S	C	S	C	S	D	O	S	DOS	DO	DS	OS
CSI	0.15 ± 0.01	0.15 ± 0.01	0.13 ± 0.01	0.14 ± 0.01	0.14 ± 0.01	0.15 0.01	0.13 ± 0.01	0.14 ± 0.01	ns	^∗^	ns	ns	ns	ns	ns
Protein	57.23 ± 4.17	51.06 ± 2.61	51.47 ± 2.24	53.57 ± 3.98	55.29 ± 3.61	52.16 ± 4.72	46.58 ± 3.76	51.51 ± 4.27	ns	ns	ns	ns	ns	ns	ns
Lipids	23.93 ± 3.31	17.83 ± 1.28	18.64 ± 2.02	21.04 ± 1.94	17.92 ± 1.24	19.63 ± 1.49	16.90 ± 1.66	18.59 ± 1.55	ns	ns	ns	ns	ns	ns	ns
Glycogen	4.35 ± 0.51	2.79 ± 0.27	4.80 ± 0.22	4.34 ± 0.40	4.12 ± 0.32	2.61 ± 0.34	4.57 ± 0.32	4.20 ± 0.56	ns	^∗∗^	^∗∗^	ns	ns	ns	ns
Total energy	2.40 ± 0.19	1.98 ± 0.92	2.25 ± 0.11	2.38 ± 0.14	2.11 ± 0.10	2.07 ± 0.16	1.87 ± 0.13	2.04 ± 0.16	ns	ns	ns	ns	ns	ns	ns

*Protein, lipids, and glycogen expressed as mg g^-1^ weight wet tissue. Total energy content expressed as kJ g^-1^ weight wet tissue. Values are mean of fish (n = 9)****±****SE. C, Control; S, Acute stress. Three-way ANOVA analysis results. D, Diet; O, dissolved oxygen levels; DOS, DO, DS, and OS, Interactions; ns, not significant P > 0.1; ^∗^P < 0.05; ^∗∗^P < 0.01.*

A decrease in IDH activity (*P* < 0.01), with an increase in LDH to IDH activity ratio (*P* < 0.05) was detected in the liver of trout reared in hypoxia (Table 4). On this tissue a decrease in LDH activity (*P* < 0.05), as well as in the LDH to IDH activity ratio (*P* < 0.01) were detected in trout fed the DEB 700 diet. However, all these metabolic parameters remained similar when acute stress was applied.

**Table 4:** Effect of dietary electrolyte balance (DEB), dissolved oxygen levels and acute stress on energy use in liver of rainbow trout.

	DEB 200				DEB 700				Factors			Interactions			
	Normoxia		Hypoxia		Normoxia		Hypoxia							
	C	S	C	S	C	S	C	S	D	O	S	DOS	DO	DS	OS
LDH	2699.12 ± 274.64	3349.83 ± 437.29	2990.73 ± 298.11	3092.40 ± 315.63	2287.73 ± 281.52	2579.46 ± 204.78	2754.66 ± 114.60	2606.28 ± 297.18	^∗^	ns	ns	ns	ns	ns	ns
IDH	84.64 ± 4.95	85.89 ± 8.59	70.13 ± 4.98	74.58 ± 4.99	94.09 ± 3.82	93.19 ± 4.51	80.05 ± 3.12	76.34 ± 4.20	ns	^∗∗^	ns	ns	ns	ns	ns
LDH/IDH	32.43 ± 3.48	39.30 ± 3.26	43.01 ± 3.18	42.69 ± 4.69	25.08 ± 3.64	28.20 ± 2.58	34.83 ± 1.96	33.56 ± 2.89	^∗∗^	^∗^	ns	ns	ns	ns	ns
Ec	211.37 ± 23.23	175.51 ± 12.78	268.48 ± 28.93	182.61 ± 22.62	183.38 ± 13.03	205.08 ± 15.22	199.70 ± 18.66	177.93 ± 22.04	ns	ns	ns	ns	ns	^∗^	ns

*Lactate dehydrogenase (LDH) and iso-citrate dehydrogenase (IDH) activities are expressed as nmol min***^-^***^1^ mg^-1^ tissue. Energy consumption value (Ec) is expressed as mJ mg^-1^ weight wet tissue. Values are mean of 9 fish****±****SEM. C, Control; S, Acute stress. Three-way ANOVA analysis results; D, Diet; O, dissolved oxygen levels; DOS, DO, DS, and OS, Interactions; ns, not significant P > 0.1; ^∗^P < 0.05; ^∗∗^P < 0.01.*

On the other hand, enzyme activities and parameters related to energy-use in the heart of trout remained similar in fish reared at different DO levels (Table 5). Both electrolyte-imbalanced diet and acute stress increased LDH to IDH activity ratio in the heart of trout (*P* < 0.05). In addition, acute stress decreased the IDH activity measured in the trout’s heart (*P* < 0.05).

**Table 5:** Effect of dietary electrolyte balance (DEB), dissolved oxygen levels and acute stress on energy use in heart of rainbow trout.

	DEB 200				DEB 700				Factors			Interactions			
	Normoxia		Hypoxia		Normoxia		Hypoxia							
	C	S	C	S	C	S	C	S	D	O	S	DOS	DO	DS	OS
LDH	596.40 ± 55.20	506.88 ± 41.59	736.26 ± 79.99	580.91 ± 53.58	596.66 ± 37.47	627.02 ± 42.02	556.57 ± 42.57	676.77 ± 45.19	ns	ns	ns	ns	ns	^∗^	ns
IDH	452.34 ± 28.98	380.32 ± 22.44	443.31 ± 23.85	427.57 ± 20.33	440.96 ± 16.12	364.53 ± 30.59	390.43 ± 19.87	407.13 ± 17.12	ns	ns	^∗^	ns	ns	ns	^∗^
LDH/IDH	1.32 ± 0.08	1.34 ± 0.08	1.52 ± 0.09	1.35 ± 0.08	1.35 ± 0.07	1.78 ± 0.12	1.42 ± 0.06	1.67 ± 0.10	^∗^	ns	^∗^	ns	ns	^∗^	ns
Ec	192.29 ± 11.73	182.52 ± 10.64	190.61 ± 22.37	160.44 ± 11.72	159.85 ± 18.85	141.11 ± 9.69	148.62 ± 9.36	208.08 ± 21.60	ns	ns	ns	^∗^	^∗^	ns	ns

*Lactate dehydrogenase (LDH) and iso-citrate dehydrogenase (IDH) activities are expressed as nmol min***^-^***^1^ mg^-1^ tissue. Energy consumption value (Ec) is expressed as mJ mg^-1^ weight wet tissue. Values are mean of fish (n = 9)****±****SE. C, Control; S, Acute stress. Three-way ANOVA analysis results. D, Diet; O, dissolved oxygen levels; DOS, DO, DS, and OS, Interactions; ns, not significant P > 0.1; ^∗^P < 0.05; ^∗∗^P < 0.01.*

### Oxidative Stress Markers in Liver

All the oxidative stress parameters analyzed in this tissue remained similar in fish reared at different DO levels (Table 6). However, in trout fed with the DEB 700 diet, a decrease in the GPX activity (*P* < 0.05), as well as an increase in the TG and GSSG levels (*P* < 0.05) were observed. In addition, acute stress induced increases in GR activity (*P* < 0.05) and GSH content (*P* < 0.05) in trout’s liver.

**Table 6:** Effect of dietary electrolyte balance (DEB), dissolved oxygen levels and acute stress on several markers of oxidative stress in liver of rainbow trout.

	DEB 200				DEB 700				Factors			Interactions			
	Normoxia		Hypoxia		Normoxia		Hypoxia							
	C	S	C	S	C	S	C	S	D	O	S	DOS	DO	DS	OS
GPx	0.64 ± 0.09	0.41 ± 0.03	0.55 ± 0.04	0.46 ± 0.03	0.34 ± 0.04	0.48 ± 0.07	0.50 ± 0.03	0.47 ± 0.08	^∗^	ns	ns	^∗^	^∗^	^∗∗^	ns
GR	3.27 ± 0.44	4.09 ± 0.33	2.56 ± 0.25	3.13 ± 0.25	3.34 ± 0.32	4.59 ± 0.57	4.11 ± 0.40	3.72 ± 0.53	ns	ns	^∗^	ns	ns	ns	ns
TG	1.14 ± 0.18	1.24 ± 0.09	1.06 ± 0.07	1.20 ± 0.07	1.28 ± 0.07	1.45 ± 0.11	1.28 ± 0.12	1.34 ± 0.13	^∗^	ns	ns	ns	ns	ns	ns
GSSG	0.53 ± 0.08	0.45 ± 0.05	0.57 ± 0.06	0.41 ± 0.07	0.55 ± 0.06	0.61 ± 0.04	0.64 ± 0.05	0.54 ± 0.04	^∗^	ns	ns	ns	ns	ns	ns
GSH	0.61 ± 0.11	0.79 ± 0.08	0.56 ± 0.06	0.79 ± 0.10	0.73 ± 0.07	0.84 ± 0.08	0.64 ± 0.10	0.86 ± 0.13	ns	ns	^∗^	ns	ns	ns	ns
LPO	5.62 ± 0.43	5.81 ± 0.52	6.38 ± 0.93	5.70 ± 0.41	5.48 ± 0.28	6.07 ± 0.79	5.28 ± 0.71	5.11 ± 0.46	ns	ns	ns	ns	ns	ns	ns

*Glutathione peroxidase (GPx) and Glutathione reductase (GR) activities in nmol min^-1^ mg prot^-1^. Total glutathione (TG), oxidized glutathione (GSSH), reduced glutathione (GSH), and lipid peroxidase (LPO) in nmol mg prot^-1^. Values are mean of fish (n = 9)****±****SE. C, Control; S, Acute stress. Three-way ANOVA analysis results. D, Diet; O, dissolved oxygen levels; DOS, DO, DS, and OS, Interactions; ns, not significant P > 0.1; ^∗^P < 0.05; ^∗∗^P < 0.01.*

### Innate Immune Status

All the parameters analyzed in plasma remained similar in fish reared at different DO levels (Table 7). However, feeding trout an electrolyte-imbalanced diet produced a decrease in ACH_50_ levels (*P* < 0.05). Acute stress increased lysozyme activity (*P* < 0.05).

**Table 7:** Effect of dietary electrolyte balance (DEB), dissolved oxygen levels and acute stress on innate immune status in plasma of rainbow trout.

	DEB 200				DEB 700				Factors			Interactions			
	Normoxia		Hypoxia		Normoxia		Hypoxia							
	C	S	C	S	C	S	C	S	D	O	S	DOS	DO	DS	OS
ACH50	13.64 ± 2.12	15.80 ± 2.28	16.25 ± 3.25	20.43 ± 2.16	10.90 ± 2.09	10.19 ± 2.20	8.59 ± 0.96	10.72 ± 1.59	^∗^	ns	ns	ns	ns	ns	ns
Lysozyme	522.22 ± 24.26	645.37 ± 44.03	633.33 ± 64.67	596.30 ± 50.29	570.37 ± 60.26	815.74 ± 138.37	544.79 ± 63.27	576.85 ± 35.05	ns	ns	^∗^	ns	ns	ns	ns
Peroxidase	116.53 ± 4.46	123.04 ± 4.87	120.26 ± 5.80	119.74 ± 4.90	126.42 ± 3.91	106.08 ± 14.32	128.59 ± 4.58	125.60 ± 2.25	ns	ns	ns	ns	ns	ns	ns

*Alternative complement pathway ACH50, Lysozyme and Peroxidase activities expressed in U mL^-1^. Values are mean of fish (n = 9)****±****SE. C, Control; S, Acute stress. Three-way ANOVA analysis results. D, Diet; O, dissolved oxygen levels; DOS, DO, DS, and OS, Interactions; ns, not significant P > 0.1; ^∗^P < 0.05; ^∗∗^P < 0.01.*

### Interactions Between Experimental Factors

No interaction between dietary treatment and DO levels was observed in parameters related to fish performance (Supplementary Table S2). In addition, no interactions were observed between the factors investigated when analyzing markers related to stress, metabolism or innate immune status in plasma (Tables 1, 7, respectively). Furthermore, the energy content of the heart of trout did not display any interaction between the experimental factors investigated (Table 3).

By contrast, the total energy content of the liver was affected by the diet and the DO levels (*P* < 0.05), as well as by the DO levels and the acute stressor (*P* < 0.01) (Table 2). The diet also influenced the energy consumption (Ec) value in the liver when the acute stressor was considered (*P* < 0.05). Additionally, the dietary treatment influenced DO levels (*P* < 0.05), acute stressor (*P* < 0.01), and all the three factors together (*P* < 0.05) when the marker of oxidative stress, GPx activity, was analyzed in liver (Table 6). The three factors together displayed significant interaction when several markers of energy use in heart of trout were investigated (LDH, IDH, LDH/IDH, Ec) (Table 5). DO levels and acute stress revealed significant interactions when glycogen content was analyzed in liver (*P* < 0.05) (Table 2).

## Discussion

This study analyzed the prolonged effects of dietary electrolyte-imbalance and environmentally low DO levels, along with an acute stressor (handling/confinement), on various functions including: (i) acute stress responses at the level of plasma or cellular stress, (ii) tissue metabolism (liver and heart), and (iii) innate immunity. By implementing such approach, the aim was to provide a refined tool to evaluate stress response in fish. The prolonged conditions implemented, DEB 700 diet and hypoxia, were designed to produce deep physiological challenges in trout. These conditions were chosen over other conditions, such as repeated physical handling, as this may increase the likelihood of skin, mucus or fin damages, and could make interpretation of chronic stress responses difficult, as this may increase the likelihood of skin, mucus or fin damages, and could make interpretation of chronic stress responses difficult due to co-factors that could be present (e.g., opportunistic pathogens), leading to erroneous conclusions.

### The Electrolyte-Imbalanced Diet Acts as a Chronic Stressor in Rainbow Trout

Growth performance and FI remains unchanged when trout was fed an electrolyte-imbalanced diet at satiety for 49 days (Supplementary Table S2). In spite of the lack of changes in these parameters, markers of energy use, oxidative stress response, and innate immune status were all altered in trout. In particular, the DEB 700 diet induced increases in HSI and total energy content in the liver of the trout. These changes could be related to an energetic constrain imposed by electrolyte-imbalanced diet, as mechanisms managing acid-base homeostasis may involve an increased energy expenditure. In fact, feeding a DEB 700 diet on this isogenic trout line has shown to produce a 65% increase in the metabolizable energy requirements for maintenance, suggesting higher energy demand to maintain acid-base balance in that group (Magnoni et al., 2018). Such scenario is supported by a postprandial decrease in blood pH of trout fed the DEB 700 diet (Table 1). An alteration on the energy balance is shown by the increase in the total energy content stored in liver of trout fed the DEB 700 diet, possibly related as well to changes in the relative importance of aerobic and anaerobic metabolism to provide energy to this organ.

As any stress condition carries additional energetic requirements, biomarkers on anaerobic and aerobic pathways of energy production may provide important indications in terms of global energetic demands toward a stress condition. In particular, LDH activity has a key role in anaerobic pathways and changes on this enzyme activity are usually associated to increased energy demand, e.g., due to stressful conditions (Dahlhoff, 2004). On the other hand, IDH, an enzyme involved in energy production through the aerobic pathways, catalyzes a key step of the Krebs cycle, and is also involved in antioxidant defenses, where it is crucial for the regeneration of NADPH required for glutathione conjugation pathways (Jo et al., 2001; Lee et al., 2002). For example, in juvenile European seabass (*Dicentrarchus labrax*) an increased IDH activity was detected in liver when exposed to high temperature, which has been linked to a stress response (Almeida et al., 2015). An increased reliance in aerobic metabolism (low LDH/IDH activities ratio) usually enables a more efficient energy metabolism than the anaerobic pathways. In our study, LDH/IDH activities ratio was decreased in liver of trout by the electrolyte-imbalanced diet. In contrast, the heart of trout displayed an increase in LDH/IDH activities ratio when fed the electrolyte-imbalanced diet, indicating that the metabolic capacity of liver and heart are modulated differently when subjected to the dietary challenge.

Key components of the glutathione system keeping redox balance, including GPX activity, TG, and GSSG levels, were all altered by diet in the liver of trout. In particular, GPx activity has been suggested as a reliable marker of stress in fish subjected to prolonged dietary or environmental challenges (Winston, 1991; Martínez-Álvarez et al., 2005; Sitjà-Bobadilla et al., 2005; Fontagné-Dicharry et al., 2018). Therefore, this study was able to confirm importance of this stress marker in the liver of trout subjected to a prolonged dietary challenge.

Haemolytic activity due to alternative complement pathway, measured as ACH_50_, is interpreted by numerous authors as a sign of a prompter innate immune system, improving the resistance to pathogens (Chiu et al., 2008; Biller-Takahashi et al., 2012). It is known that ACH_50_ activity in fish changes with stress conditions (Boshra et al., 2006). Furthermore, decreased ACH_50_ activity has been linked to chronic stress produced by crowding in gilthead seabream (*Sparus aurata*) juveniles (Montero et al., 1999b). Trout fed the DEB 700 diet displayed a decreased ACH_50_ activity in plasma, which reinforces the conclusion that the disruption on fish homeostasis generated by the electrolyte-imbalanced diet could be evoking a stress response. Fish are able to mount successful and robust innate responses (Tort et al., 2003), such as the measured in this study. Previous studies have shown that the innate immune response in fish is altered by chronic crowding stress (Rotllant et al., 1997; Montero et al., 1999a) and by a combination of dietary supplementations and chronic crowding stress (Montero et al., 1999b). However, future studies should investigate the response of adaptive immune parameters to combined stressors such as those implemented in this study.

### Rainbow Trout Shows a Full Adaptive Response to Chronic Hypoxia

Hypoxia tolerance appears to be variable in close phylogenetic related species of fish (Mandic et al., 2009), also displaying marked changes during development (Ishibashi et al., 2005). In this sense, the heterozygous isogenic line of rainbow trout used in this study, reared for many generations, provides a unique model with low intraspecific variability, adequate to test the effect of chronic hypoxia. This study is a follow-up of a previous one (Magnoni et al., 2018), in which it was shown that both feed intake (FI) and growth performance were decreased when this trout line was exposed to chronic hypoxia. In particular, rainbow trout maintained under hypoxia (4.5 ± 0.1 mg O_2_ L^-1^) reduced their feed intake and specific growth rate by 33 and 22%, respectively (Supplementary Table S2). In a previous study, Glencross (2009) also showed a decrease in FI in rainbow trout exposed to hypoxia (28 days at 4.29 mg O_2_ L^-1^). Therefore, the decrease in FI during chronic hypoxia detected in the present study is probably not linked to chronic stress, but more likely to a reduction in appetite triggered by the inhibition of food-anticipatory behavior, as has been suggested by previous studies (Glencross, 2009; Folkedal et al., 2012a,b). Corticotropin-releasing factor in the forebrain appears to mediate sustained anorexia during chronic hypoxia (Bernier and Craig, 2005), suggesting that this mechanism may be an essential energy-saving strategy due to the reduction in fish maintenance energy. Also suppression of appetite may save energy by reducing the postprandial increase in O_2_ consumption associated with digestion, absorption, transformation, and storage of nutrients (Jobling, 1981, 1993). In this study, we cannot completely rule out the possibility that chronic hypoxia could act as a chronic stressor in trout, inhibiting feed intake by altering feeding behavior. However, as will be discussed in the next section, we propose that this may not be the case for this study, since the cortisol response was not altered after chronic low DO levels, suggesting that trout is adapted to these particular conditions.

There is increasing evidence that rearing fish under intensive aquaculture conditions may alter their cardiovascular physiology (Gamperl and Farrell, 2004). The DO level applied in the hypoxia treatment during this study (4.5 mg O_2_ L^-1^) is recognized as an environmental challenge, with the value decided based on the critical DO level for feed consumption and for both growth rate and feed conversion efficiency (6 and 7 mg O_2_ L^-1^, respectively) for rainbow trout (Pedersen, 1987). However, a previous study showed that rainbow trout reared at hatcheries, with sub-optimal water quality conditions, appear to have a remarkable degree of tolerance to short-term severe hypoxia, which has been linked to advantageous cardiovascular adaptations to enhance the animal’s function (Faust et al., 2004). Previous studies have shown that chronic hypoxia in both zebrafish (*Danio rerio*) and the cichlid *Haplochromis piceatus* lead to decreases in the ventricular space, suggesting a profound restructuration of this organ (Marques et al., 2008). Our results also suggest that the trout population used in the trial displayed a good capacity to adapt to the chronic hypoxic conditions. In particular, the rainbow trout subjected to 49 days of hypoxia displayed a reduction in heart to body mass index (CSI), but increased the glycogen content compared to the normoxic group. Therefore, the rainbow trout isogenic line appears to have the capacity to fully adapt to the applied hypoxic levels, which was confirmed by the lack of significant change in all the markers of oxidative stress (liver) and innate immune status (plasma) markers investigated on this study.

### Reliability of Markers for Acute and Chronic Stress

Changes in plasma cortisol levels are commonly used to assess acute stress conditions in fish, as a peak in the release of this hormone is associated with stress response. As expected, in this study, the cortisol levels in plasma of trout subjected to acute stressor were significantly increased when compared to their controls. However, the utility of changes in the levels of this cortisol to evaluate chronic stress in fish is not always possible due to several mechanisms reducing cortisol levels, including negative feedback (Pickering and Stewart, 1984; Mommsen et al., 1999) and the increased metabolic clearance rate of cortisol, all mechanisms which overall result in suppression of the cortisol response (see reviews Wendelaar Bonga, 1997; Gorissen and Flik, 2016). Thus, in rainbow and brown trout, chronic confinement stress for 6 weeks resulted in an elevation of plasma cortisol levels up to 4 weeks before returning to basal level after 42 days (Pickering and Pottinger, 1989; Person-Le Ruyet et al., 2008). A similar response was observed in Atlantic salmon parr or smolt exposed to repeated chasing stress over 3 weeks, when a decline in plasma cortisol levels was observed after 1 week, suggesting a rapid habituation/desensibilization of the HPI axis (Madaro et al., 2016). However, cortisol response to chronic stressors may vary according to species and/or the type of stressor. For example, seabass exposed for 57 days to water quality deterioration did not show any significant changes in the basal cortisol levels (Santos et al., 2010), whereas in other study in seabream and seabass, the same parameter increased after 3 weeks when exposed to three levels of chronic stress (confinement/chasing/air exposure) (Samaras et al., 2018). Another approach to characterize chronic stress status is the analysis of cortisol response to acute confinement stress, which affects the HPI axis responsiveness. This approach has been implemented in rainbow trout and salmon by applying chronic crowding stress or repeated acute stress over several weeks, resulting in a less pronounced cortisol response to acute stress (Madaro et al., 2015, 2016; Moltesen et al., 2016). In contrast, seabass exposed to water quality deterioration showed an increased cortisol response to acute stress (Santos et al., 2010) and a similar response was also observed in trout exposed to chronic hypoxia (Leguen and Prunet, unpublished). Overall, this data indicates that chronic stress generally leads to dysregulation of cortisol production (basal and/or acute stress levels) which varies according to species and stressor. In the present study, exposure of rainbow trout to chronic low DO levels did not modify cortisol levels nor cortisol response, which fits with our previous conclusions that indicate fish were adapted to these hypoxic conditions.

In this study, the prolonged dietary challenge increased the cortisol levels in plasma of trout 1.23 times. However, such increase was modest compared to the spike of 4.08 times in plasma cortisol levels of trout when subjected to the acute stressor. In spite of these observed changes, cortisol levels were not significantly different in trout fed the diets with different DEB and then subjected to acute stress (*P* < 0.057), also indicated by the lack of interactions between both factors (*P* = 0.284). These results may suggest that the capacity of trout to mount a proper cortisol response when subjected to an acute stressor remained unaltered by the dietary treatment. These results contrast with studies presented in the previous paragraph where chronic stress leads to a clear dysregulation of cortisol responsiveness in fish. Therefore, additional studies are required to further appraise how the cortisol response and the allostatic load of trout may be altered under chronic stressors, for example when subjected to dietary challenges.

No detectable changes were observed in LPO, a marker of oxidative damage, in the liver of trout exposed to the experimental conditions, even when facing the acute stressor. However, components of the glutathione system (GR and GSH) were increased in liver of trout subjected to the acute stressor, indicating an alteration on the balance between prooxidants/antioxidants in this organ. In addition, another component of the glutathione system, the GPx activity in liver, displayed an interaction between dietary treatment and acute stress, as well as for all three factors together (*P* < 0.01), suggesting that the prolonged dietary challenge places a constraint in trout exposed to a subsequent acute stressor.

The innate immune system in fish is constitutive, reacting at short time scale to non-specific cues and induced by several external molecules (Tort et al., 2003; Tort, 2011). In particular, lysozyme activity is used as an indicator of non-specific immune response (Tort et al., 1996, 1998), rapidly induced in fish exposed to acute stressors (Demers and Bayne, 1997; Rotllant and Tort, 1997). Although lysozyme activity can vary considerably between fish species, this enzymatic activity in plasma appears to be modulated in response to both physical and nutritional cues (Möck and Peters, 1990; Montero et al., 1999b). The increased lysozyme activity in plasma of trout subjected to acute stress observed in this study suggest a link between stress and suppression of the innate immune response, which has important implications for aquaculture. As it was suggested earlier by Fevolden et al. (1999), it is possible to hypothesize that the enhanced lysozyme activity after exposure to the acute stressor may not be directly related to pathogen resistance, and may rather reflect disease-susceptible fish. In this study ACH_50_ activity was decreased by the dietary imbalance, where lysozyme activity was decreased by the acute stressor in plasma of rainbow trout. Surprisingly, no interaction between the two factors was detected for any of the innate immune status parameters analyzed. Therefore, it will be necessary to further investigate how chronic stressors may be affecting not only the innate but also the adaptive immune response of rainbow trout exposed to acute challenges.

**FIGURE 1:**
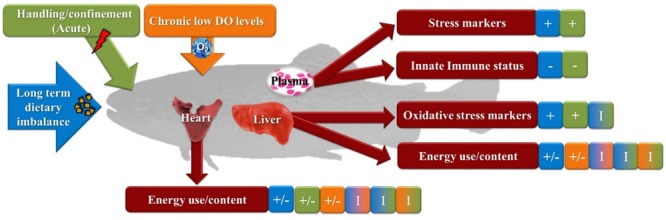
Summarized output of biomarkers studied in rainbow trout exposed to a combination of chronic and acute conditions. Fish were fed a balanced- or an electrolyte-imbalanced diet (DEB 200 or DEB 700, respectively) and reared at different environmental oxygen levels (normoxia or hypoxia) for 49 days. Subsequently fish were subjected to a standard acute stress protocol (2 min of handling/confinement) and compared to undisturbed group (control). +, increased by the condition. -, decreased by the condition. I, Interactions.

A summarized description of the results obtained from this study is presented in Figure 1. This study suggest that the trout isogenic line displays a complete adaptive response to chronic hypoxia, whereas the challenge imposed by feeding an electrolyte-imbalanced diet profoundly affected fish homeostasis after 49 days. Such physiological condition in fish appear to lead to stress response as indicated by several markers of oxidative stress and decreased innate immune response. Although no interactions in the markers for innate immune response were detected between the different factors analyzed, the dietary challenge, by feeding trout the DEB 700 diet, suggests an impairment of the physiological response in this fish to a subsequent acute stressor. This is shown in this study by an increase in the levels of several oxidative stress markers detected in liver of trout exposed to the acute stressor in trout fed the electrolyte-imbalance diet. Nevertheless, the results presented here were obtained in an isogenic line and may not necessarily reflect the response from other rainbow trout used in aquaculture.

## Conclusion

In conclusion, this study suggests that dietary imbalances present during rearing may act as chronic stressors, interacting with other well-established stressors such as the acute stressors applied on this study. These results are of great interest to the aquaculture industry as both of these factors may be present in cultured fish and could be linked to negative effects. Future studies should further investigate the physiological and behavioral aspects associated to such stress responses, and how to reduce their occurrence.

## Ethics Statement

The fish trials were approved and carried out according to the Wageningen University Ethics Board for experimentation with animals (DEC, Registration protocol 2014056.a), under Dutch and EU legislation on the handling of experimental animals.

## Author Contributions

LM, IG, PP, and JS conceptualized the study. LM, SN, EE, ML, RO, IL, PP, IG, and JS were involved in the methodology and formal analysis. LM, SN, PP, and JS were involved in writing the original draft. All authors reviewed the manuscript.

## Conflict of Interest Statement

The authors declare that the research was conducted in the absence of any commercial or financial relationships that could be construed as a potential conflict of interest.
