# Supplementary material for: Acute Stress and an Electrolyte- Imbalanced Diet, but Not Chronic Hypoxia, Increase Oxidative Stress and Hamper Innate Immune Status in a Rainbow Trout (Oncorhynchus mykiss) Isogenic Line
Source: Front Physiol. 2019 Apr 24;10:453. doi: 10.3389/fphys.2019.00453 (PMC6491711; doi:10.3389/fphys.2019.00453)
Supplement: Supplementary file 1 [file Table_1.DOCX]

**Table S1**. Ingredients and proximate composition of the experimental diets.

| Test ingredients (%) | DEB 200 | DEB 700 |
| --- | --- | --- |
| Na_2_CO_3_ | 0.3 | 2.9 |
| Diamol* | 2.7 | 0.1 |
| Wheat | 27.2 | 27.2 |
| Wheat gluten | 13.0 | 13.0 |
| Fish meal^¶^ | 13.0 | 13.0 |
| Fish oil^&^ | 14.0 | 14.0 |
| Soya protein concentrate | 13.0 | 13.0 |
| Pea protein concentrate | 13.0 | 13.0 |
| Lysine HCL | 0.3 | 0.3 |
| DL-methionine | 0.5 | 0.5 |
| Monocalcium phosphate | 1.5 | 1.5 |
| CaCO_3_ (krijt) | 0.5 | 0.5 |
| Yttrium oxide | 0.01 | 0.01 |
| Premix^£^ | 1.0 | 1.0 |
| Total | 100.0 | 100.0 |
| Proximate composition |  |  |
| DM (%) | 93.0 ± 0.1 | 92.6 ± 0.2 |
| Ash (% on DM) | 11.2 ± 0.1 | 10.6 ± 1.2 |
| Crude protein (% on DM) | 45.0 ± 0.1 | 45.2 ± 0.3 |
| Crude lipid (% on DM) | 15.3 ± 0.2 | 16.0 ± 0.2 |
| TC (% on DM) | 31.6 ± 0.2 | 31.5 ± 0.1 |
| GE (kJ g DM^-1^) | 22.0 ± 0.1 | 22.4 ± 0.2 |

DEB, dietary electrolyte balance (mEq kg^-1^). *Diamol GM; Franz Bertram. ^¶^ RE>680.

^£^ Vitamin premix composition (to supply, mg/kg feed): 10, B1; 10, B2; 20, B3; 40, B5; 10, B6; 0.2, biotin; 2, folic acid; 0.015, B12; 2000, choline (as choline chloride); 100, C (as ascorbic acid C phosphate); 3000 IU, A (as A palmitate), 2400 IU, cholecalciferol (Rovimixw D3-500; DSM, Inc.); 100 IU, E; 10, menadione (as menadione sodium bisulfite, 51 %); 400, inositol; 100, antioxidant BHT (E300-321); 1000, calcium propionate. Mineral premix composition (to supply, mg/kg feed): 50, Fe (as FeSO_4_.7H_2_O); 30, Zn (as ZnSO_4_.7H_2_O); 0.1, Co (as CoSO_4_.7H_2_O); 10, Cu (as CuSO_4_.5H_2_O); 0.5, Se (as Na2SeO3); 20, Mn (as MnSO_4_.4H_2_O); 500, Mg (as MgSO_4_.7H_2_O); 1, Cr (as CrCl_3_.6H_2_O); 2, I (as CaIO_3_.6H_2_O). DM, dry matter; TC, total carbohydrates; GE, gross energy. Proximate composition values are presented as mean±SEM (n=3). No statistical differences were found for any of parameters analysed between the diets (P> 0.05).
