# Supplementary material for: Acute Stress and an Electrolyte- Imbalanced Diet, but Not Chronic Hypoxia, Increase Oxidative Stress and Hamper Innate Immune Status in a Rainbow Trout (Oncorhynchus mykiss) Isogenic Line
Source: Front Physiol. 2019 Apr 24;10:453. doi: 10.3389/fphys.2019.00453 (PMC6491711; doi:10.3389/fphys.2019.00453)
Supplement: Supplementary file 2 [file Table_2.DOCX]

**Table S2.** Effect of dietary electrolyte balance (DEB) and dissolved oxygen levels on rainbow trout performance.

|  | **DEB 200** | | **DEB 700** | | **Factors** | | |
| --- | --- | --- | --- | --- | --- | --- | --- |
|  | **Normoxia** | **Hypoxia** | **Normoxia** | **Hypoxia** | **D** | **O** | **I** |
| **BW_f_** | 207.38 ± 9.57 | 185.33 ± 6.74 | 214.54 ± 5.92 | 186.95 ± 4.82 | ns | ** | ns |
| **FI** | 1.61 ± 0.19 | 1.09 ± 0.01 | 1.74 ± 0.23 | 1.16 ± 0.04 | ns | * | ns |
| **FCR** | 0.76 ± 0.01 | 0.77 ± 0.02 | 0.79 ± 0.01 | 0.81 ± 0.01 | * | ns | ns |
| **SGR** | 1.29 ± 0.09 | 0.97 ± 0.04 | 1.35 ± 0.14 | 0.96 ± 0.02 | ns | * | ns |

BW_f_= Final body weight in g. FI= feed intake in g DM fish^-1^ day^-1^. FCR= fed conversion ratio. SGR= specific growth rate in % weight day^-1^. Values are mean per tank (n=3) ± SEM. Two-way ANOVA analysis results. D= Diet; O= dissolved oxygen levels; I = Interaction D x O. ns= not significant P>0.1; *P<0.05; **P<0.01.
